# Supplementary material for: Levels of high-sensitive troponin T and mid-regional pro-adrenomedullin after COVID-19 vaccination in vulnerable groups: monitoring cardiovascular safety of COVID-19 vaccination
Source: Front Cardiovasc Med. 2024 Oct 18;11:1435038. doi: 10.3389/fcvm.2024.1435038 (PMC11527644; doi:10.3389/fcvm.2024.1435038)
Supplement: Supplementary file 1 [file Datasheet1.pdf]

Supplementary Material

# Levels of high-sensitive troponin T and mid-regional pro-adrenomedullin after COVID-19 vaccination in vulnerable groups: a prospective study on subtle and persistent cardiovascular involvement

**Samipa Pudasaini[1], Ngoc Han Le[2], Dörte Huscher[3], Fabian Holert[1], David Hillus[2], Pinkus Tober-Lau[2], Florian Kurth[2], Leif Erik Sander[2, 4†], Martin Möckel[1*†]**

*** Correspondence:**
Prof. Dr. med. Martin Möckel
martin.moeckel@charite.de

## 1. Supplementary Figures and Tables

### 1.1. Supplementary Figures

**Supplementary Figure 1.**

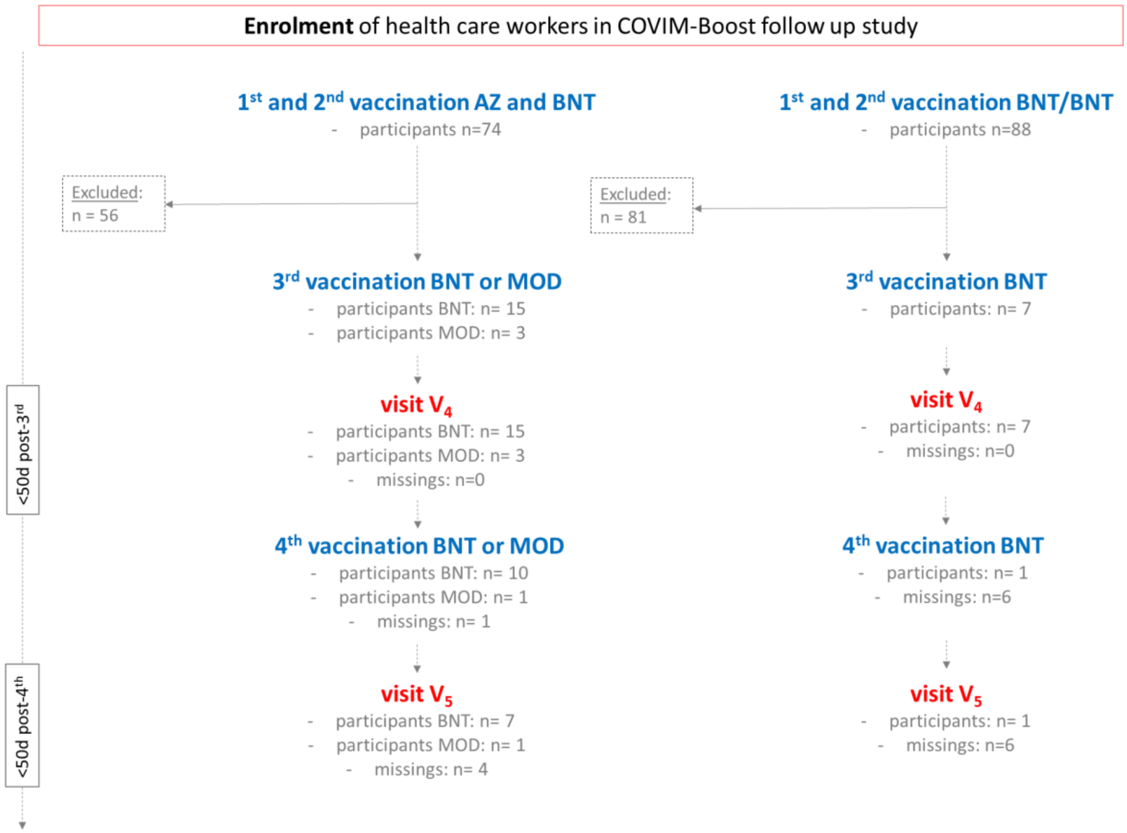


Study population diagram of the follow-up study COVIM-Boost. A 3rd (and optionally 4th) vaccination was done and visit data was collected at $V_4$ and $V_5$.

Abbreviations: BNT BNT162b2 messenger ribonucleic acid vaccine from BioNTech, MOD messenger ribonucleic acid -1273 vaccine from Moderna, $V_1$-$V_5$ visiting times 1-5, w week(s).

**Supplementary figure 2. A.** HsTnT values at all six visiting points (including the follow ups) for participants of the AZ/BNT and BNT/BNT group (HCWs and elderly), separated by sex.

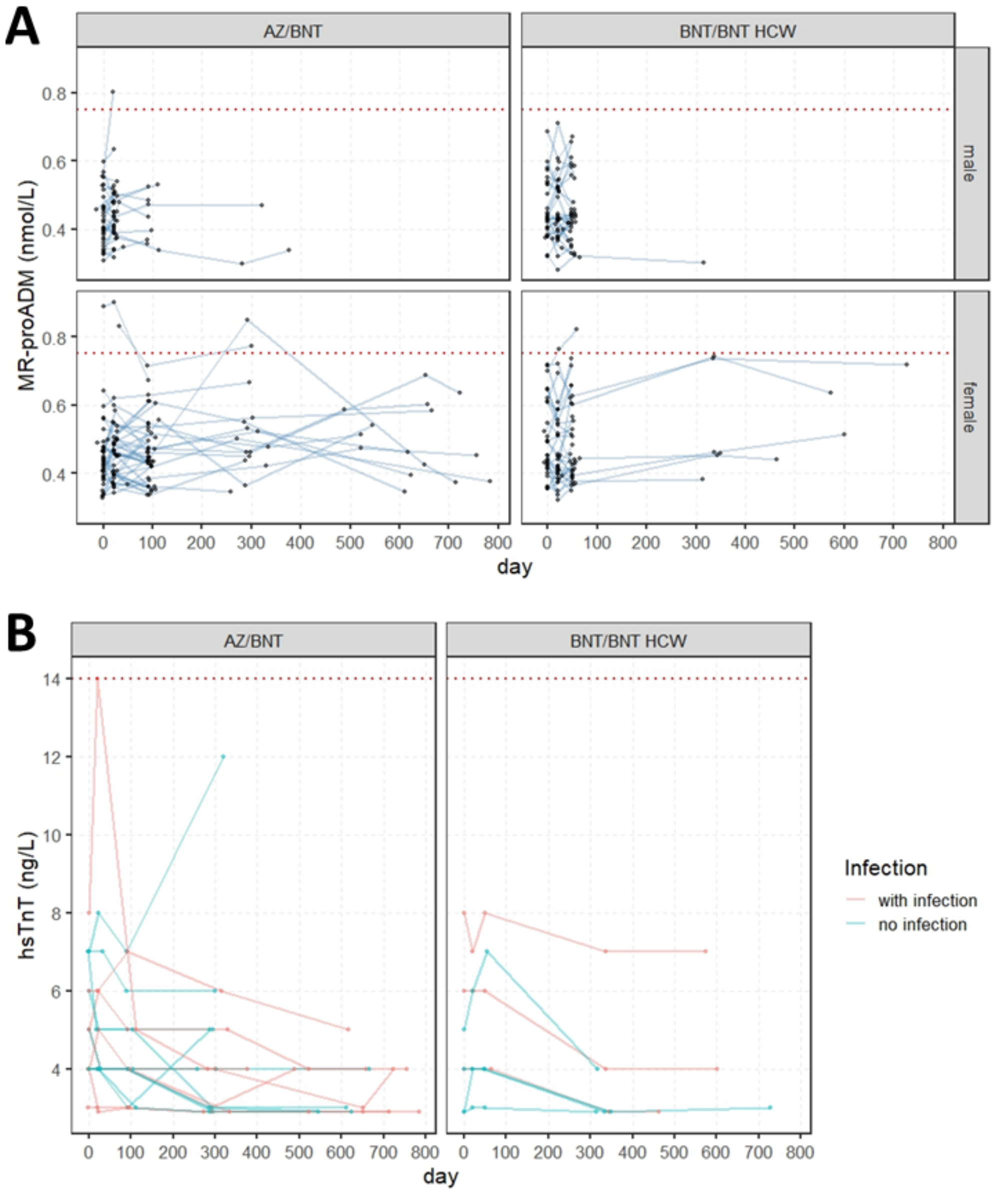


The red dashed line indicates the 14 ng/L threshold. **B.** Box plots of the change of hsTnT values between each visiting time ($V_1$-$V_2$, $V_1$-$V_3$, $V_1$-$V_3$).

Abbreviations: AZ ChAdOx1 nCov-19 adenoviral vector vaccine from Astra Zeneca, BNT BNT162b2 messenger ribonucleic acid vaccine from BioNTech, HCW health care workers, hsTnT high-sensitive troponin T.

**Supplementary figure 3.** MR-proADM values at the five visiting points ($V_1$-$V_5$) for participants of the AZ/BNT and BNT/BNT group (including HCWs and elderly), separated by sex.

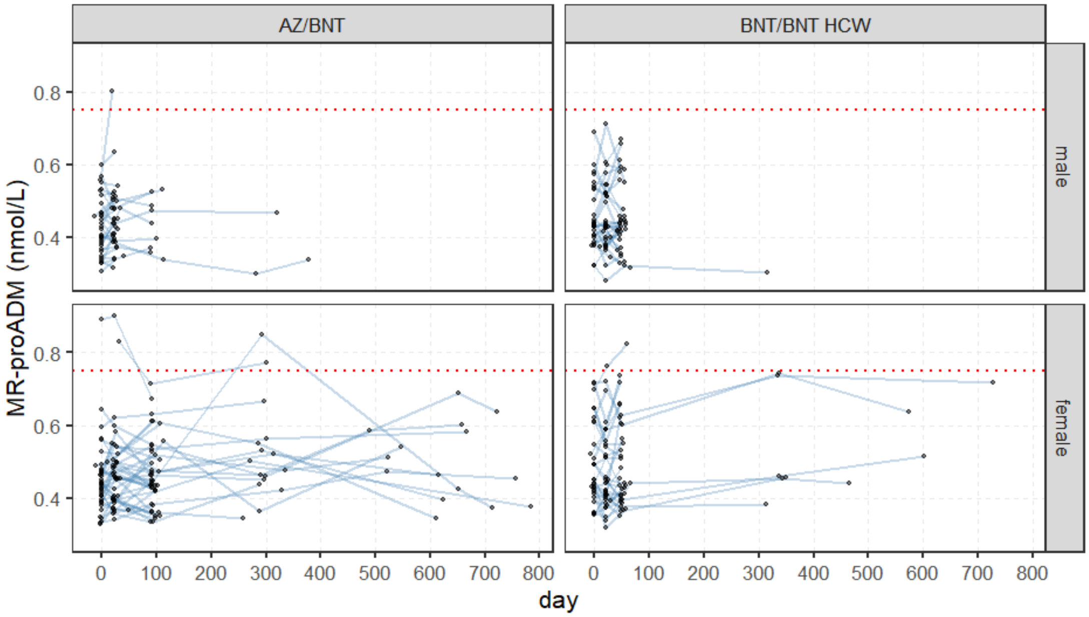


The red dashed line indicates the 0.75 nmol/L threshold.

<u>Abbreviations</u>: AZ ChAdOx1 nCov-19 adenoviral vector vaccine from Astra Zeneca, BNT BNT162b2 messenger ribonucleic acid vaccine from BioNTech, MR-proADM mid-regional pro-adrenomedullin, $V_1$-$V_5$ visiting times 1-5.

**Supplementary figure 4.** The correlation scatter plots are presented separately for sex with Spearman correlation coefficients. **A)** Correlation between hsTnT and MR-proADM values at all 3 visits. **B)** Correlation between age and MR-proADM levels at $V_1$. **C)** Correlation between age and hsTnT levels at $V_1$.

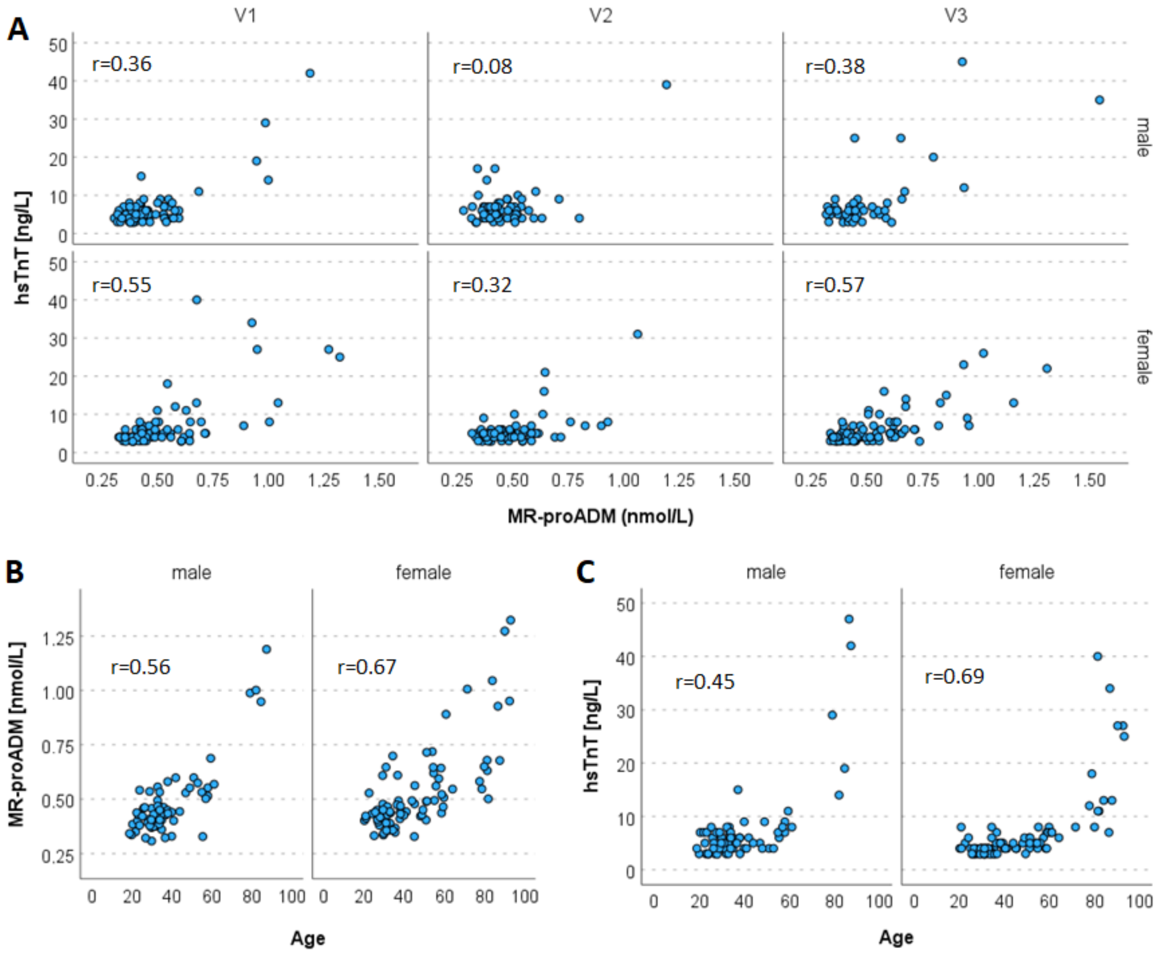


Abbreviations: AZ ChAdOx1 nCov-19 adenoviral vector vaccine from Astra Zeneca, BNT BNT162b2 messenger ribonucleic acid vaccine from BioNTech, hsTnT high-sensitive troponin T, MR-proADM mid-regional pro-adrenomedullin.

**Supplementary Table 1.**

| | COVIM cohort HCW | Selection HCW | P value | COVIM cohort Seniors | Selection Senior | P Value |
|---|---|---|---|---|---|---|
| Numbers (n) | 335 | 142 | | 311 | 20 | |
| *Patient characteristics* | | | | | | |
| Age, *years* # | 34.0 (29.0, 45.0) | 34.0 (30.0, 45.0) | 0.80 | 80.0 (77.0, 85.0) | 83.7 (80.7, 87.0) | 0.05 |
| Sex, *female* | 201/335 (60%) | 75/142 (53%) | 0.18 | 215/311 (69%) | 15/20 (75%) | 0.76 |
| BMI, *kg/m* $^{2}$ # | 23.73 (21.50, 26.17) n=321 | 23.82 (21.51, 25.95) n=141 | 0.80 | 24.51 (22.22, 27.31) n=236 | 23.2 (21.5, 26.0) | 0.13 |
| Former smokers | 20/320 (6%) | 6/128 (5%) | 0.68 | 54/311 (17.4%) | 0/20 (0%) | 0.08 |
| Current smokers | 43/320 (13%) | 9/128 (7%) | 0.08 | 13/311 (4.2%) | 1/20 (5%) | 1 |
| *Comorbidities* | | | | | | |
| Cardiovascular diseases | 29/335 (8,7%) | 7/142 (0.5%) | 0.22 | 261/311 (83.9%) | 14/20 (70%) | 0.19 |
| Hypertonus | 27/335 (8%) | 7/142 (0.5%) | 0.31 | 215/311 (69.1%) | 13/20 (65%) | 0.89 |
| Heart insufficiency | 1/335 (0.3%) | 0 (0.0%) | 1 | 36/311 (11.6%) | 4/20 (20%) | 0.44 |
| Heart rhythm disorder | 1/335 (0.3%) | 0 (0.0%) | 1 | 82/311 (26.4%) | 3/20 (15%) | 0.39 |
| Myocardial infarction, Angina pectoris, peripheral artery disease, carotid stenosis | 1/335 (0.3%) | 0 (0.0%) | 1 | 42/311 (13.5%) | 3/20 (15%) | 1 |
| Chronic lung diseases | 31/335 (9.3%) | 8/142 (6%) | 0.26 | 78/311 (25.1%) | 2/20 (10%) | 0.21 |
| Kidney diseases | 2/335 (0.6%) | 1/142 (0%) | 1 | 64/311 (20.6%) | 6/20 (30%) | 0.47 |
| Cancer | 8/335 (2.4%) | 4/142 (0%) | 1 | 86/311 (27.7%) | 3/20 (15%) | 0.33 |

| | | | | | | |
|---|---|---|---|---|---|---|
| *Premedication* | | | | | | |
| Immunosuppression/ Antihypertensive drugs | 9/335 (2%) | 5/142 (4%) | 0.84 | 232/311 (75%) | 13/20 (65%) | 0.49 |
| *SARS-CoV-2 Serology* | | | | | | |
| SeraSpot spike V1[1#] | 0 (0, 0.02) n=309 | 0 (0,0) n=134 | 0.09 | 0 (0,0) n=103 | 0 (0,0) n=16 | 0.74 |
| SeraSpot N reactive[2] V1 (n/N %) | 4/309 (1%) | 0/134 (0%) | 0.44 | 6/103 (7%) | 0/16 (0%) | 0.71 |
| SeraSpot spike V2[#] | 1.57 (0.62, 2.41) n=280 | 1.58 (0.62, 2.47) n=141 | 0.87 | 0.38 (0.1, 1.28) n=95 | 0.23 (0.06, 1.37) n=17 | 0.24 |
| SeraSpot N reactive V2 (n/N %) | 7/280 (2.5%) | 0/141 (0%) | 0.14 | 5/95 (5%) | 0/17 (0%) | 0.74 |
| SeraSpot spike V3[#] | 4.95 (4.38, 5.62) n=290 | 5.05 (2.59, 4.51) n=141 | 0.67 | 3.81 (2.84, 4.97) n=128 | 3.03 (2.18, 4.75) | 0.13 |
| SeraSpot N reactive V3 (n/N %) | 10/290 (3%) | 0/141 (0%) | 0.06 | 4/128 (3%) | 0/20 (0%) | 0.95 |

#: median with interquartile range (IQR). N.a.: not applicable.

[1] Measured in signal to cutoff ratio [S/co]

[2] Reactive is defined as >1.0 COI

**Supplementary Table 1.** The supplementary table compares patient characteristics, comorbidities, premedication and SARS-CoV-2 serology of the selection cohort for our investigation with the overall EICOV/COVIMMUNIZE/COVIM cohort. Statistical analyses were performed with Mann-Whitney U-Test and Pearson's Chi-squared test with Ytes' continuity correction. No significance was shown between the selection groups and the overall cohort.
